# Overcoming Hurdles in Hepatitis C Virus Research: Efficient Production of Infectious Virus in Cell Culture

**Published:** 2008-06

**Authors:** Erica Silberstein, Deborah R. Taylor

**Affiliations:** *Laboratory of Hepatitis and Related Emerging Agents, Center for Biologics Evaluation and Research, US Food and Drug Administration*

**Keywords:** hepatitis C virus, cell culture systems, replicons, particle assembly, viral spread and replication

## Abstract

Hepatitis C virus is a flavivirus that infects nearly 2% of the world population. There is no vaccine available and current therapy with interferon and ribavirin is expensive, not well tolerated and effective in only 60% of patients. HCV research has been hampered by the lack of a robust tissue culture system, but recent advances have made virus growth in culture possible. Here we review the current state-of-the-art and the molecular hurdles that have been met and those that still need to be overcome.

## INTRODUCTION

Hepatitis C virus (HCV) is an enveloped, positive-sense RNA virus of the *Flaviviridae* family that causes acute and chronic liver diseases (1). Six major genotypes have been identified for HCV that are further divided into numerous subtypes (2). The high mutation rate of the RNA genome generates viral diversity that leads to the existence of multiple quasispecies within an infected individual (3). About 170 million people are infected with HCV worldwide and these individuals are at high risk of developing cirrhosis and hepatocellular carcinoma.

Currently, there is no vaccine for HCV. Recombinant vaccine candidates have proved largely unsuccessful in mounting a protective response in chimpanzees. Combination therapy utilizing interferon alpha (IFN-α) and ribavirin is successful in only half of the patients (4). A major obstacle in the development of effective vaccines and improved therapeutics has been the lack of a reproducible and efficient tissue culture system for propagation of HCV (5).

## GENETIC ORGANIZATION AND LIFE CYCLE OF HCV

**Figure 1:**
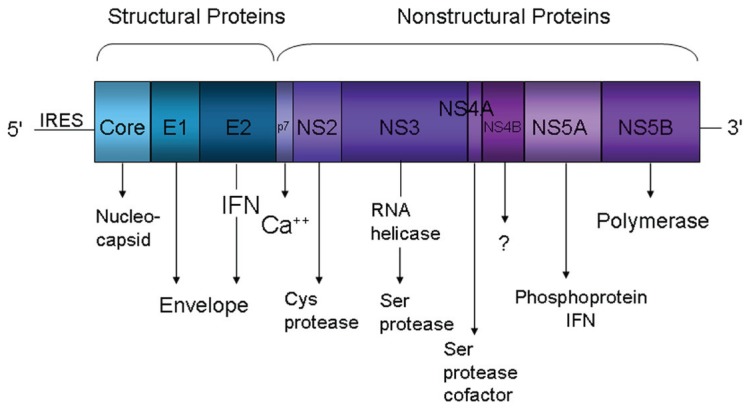
**HCV genome organization.** The HCV genome encodes a single large open reading frame that is flanked by 5’ and 3’ non-coding regions (NCR). It is translated into a single polyprotein by the activity of an IRES element located at the 5’ NCR. The polyprotein is cleaved by viral and cellular proteases into 10 polypeptides including three structural and seven nonstructural (NS) proteins.

HCV is a small enveloped positive-strand RNA virus of the family *Flaviviridae*. The 9,600 nucleotides genome encodes a single large polyprotein that is post-translationally cleaved by viral and cellular proteases into 10 polypeptides; including three structural (core and the envelope glycoproteins E1 and E2) and seven nonstructural (NS) proteins (p7 and NS2, 3, 4A, 4B, NS5A and NS5B) (Figure 1). The structural proteins participate in viral entry and in the assembly of new particles, while the non-structural proteins play important roles in replication, assembly, and pathogenesis (6).

**Figure 2:**
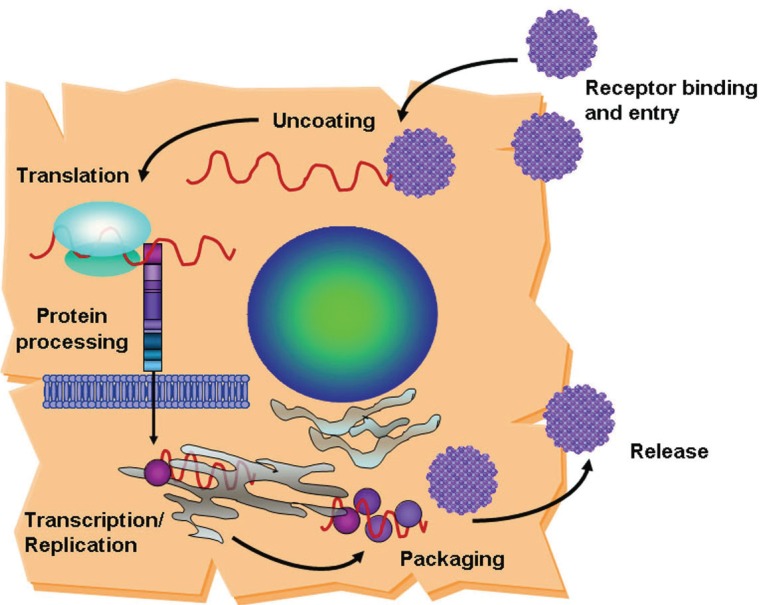
HCV life cycle. After entry into the cell and uncoating, the HCV RNA undergoes translation, protein processing, replication and packaging into nascent virions, which are transported out of the cell.

HCV is internalized in cells by receptor-mediated endocytosis. Several cellular co-receptors associated with lipid and lipoprotein metabolism have been proposed to mediate HCV entry into cells. CD81(7-9), the scavenger receptor class B type I (SR-BI) (10, 11) and claudin-1 (12) are likely to be specific and have been demonstrated as required for entry. Other cellular surface molecules have also been implicated in HCV entry: low density lipoprotein receptor (13, 14), dendritic-cell-specific intercellular adhesion molecule 3-grabbing non-integrin (DC-SIGN) (15-17) and Liver/lymph node-specific intercellular adhesion molecule-3-grabbing integrin (L-SIGN) (18, 19). Nevertheless, cells expressing all of the putative co-receptors are still resistant to HCV, indicating the need of additional factors for viral entry (20). Binding of HCV to its receptor leads to fusion between the virion envelope and the cell membrane and release of the viral RNA into the cytoplasm of the host cell (Figure 2). The genome serves as a messenger RNA (mRNA) for translation of the viral proteins and constitutes the template for RNA replication. Translation depends on an internal ribosome entry site (IRES), located partially within the 5’-noncoding region (NCR), which binds to the host 40s ribosomal subunit without the need of most cell translation initiation factors (Figure 1) (21). The single viral polyprotein is cleaved by host signal peptidases and subsequently by viral proteases into the mature proteins. The HCV polymerase (NS5B) copies the positive-strand viral RNA into full-length anti-sense strand RNA, which becomes the template for both mRNA and genomic RNA. The full-length positive-sense RNA is packaged into the viral particles containing the core and envelope proteins. Virions presumably form by budding into the endoplasmic reticulum (ER) and are exported through the cell secretory pathway (22).

## FIRST ATTEMPTS AT HCV CELL CULTURE

Propagation of HCV has been extremely difficult. Before the identification of HCV as the causative agent behind non-A, non-B hepatitis infections, few infection systems were reported before 1999, and they all demonstrated low reproducibility and efficiency (5). These studies were initially performed using primary hepatocytes from humans or chimpanzees. Human fetal hepatocytes were successfully infected with HCV; however the system supported HCV replication that was only detectable by RT-PCR amplification (23). It was also shown that adult primary human hepatocytes could be infected and supported long-term culture for up to 4 months (24).

In addition, HCV was adapted to grow in the human T- and B-cell lines HPBMa10-2 and Daudi (25). Infectious virus was recovered from these cultures more than one year after infection. A model for chronic infection of suckling mouse brain (SMB) cells actively producing the virus was established by Deryabin *et al*. (26). They reported that persistent HCV contained in the SMB cultures induced a cytopathogenic effect in several cell lines, but these results have not be reproduced by other laboratories as of yet.

## THE REPLICON AGE: DEVELOPMENT OF CELL-BASED REPLICATION SYSTEMS

**Figure 3:**
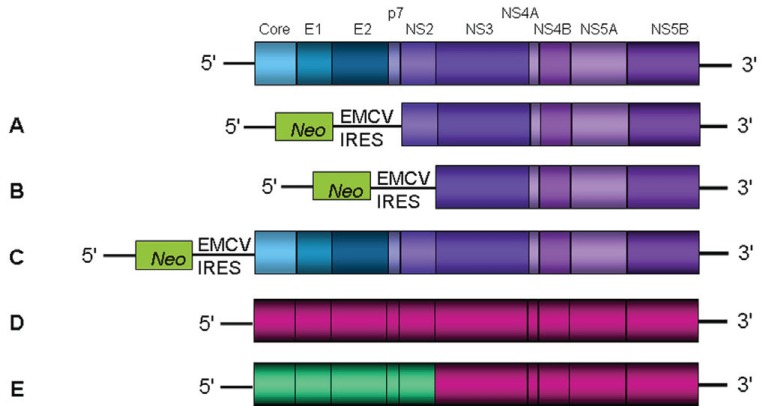
HCV Replicon Systems. HCV genes are shown (top). (**A, B**) Subgenomic replicons (27, 28); (**C**) Full-length replicon (38); (**D**) JFH-1 (58); (**E**) chimeric replicon J6/JFH-1 (55).

The development of HCV replicons in 1999 constituted a major advance and allowed the study of RNA replication and virus-cell interactions in the human hepatoma cell line Huh-7. The source of the first subgenomic HCV bicistronic replicon was total RNA isolated from a patient with chronic hepatitis C infection (27). Using long distance RT-PCR, the complete open reading frame was amplified in two overlapping fragments. After analysis of several clones of each fragment, an isolate-specific consensus sequence of genotype 1b was established. This replicon contains the HCV 5’ noncoding region fused to the first 12 amino acids of the core protein, the neomycin phosphotransferase coding region (*Neo*), which confers resistance to the antibiotic geneticin, regulated by the internal ribosomal entry site (IRES) of HCV. The encephalomyocarditis virus (EMCV) IRES drives translation of the HCV non-structural proteins, and the HCV 3’ noncoding region (Figure 3A, B). After transfection of Huh-7 cells with RNA transcribed *in vitro* by T7 RNA polymerase from the linearized replicon plasmid, cells were selected by culturing them in the presence of geneticin. These cells maintained the viral “subgenomes”, but only a limited amount of geneticin-resistant colonies were obtained. Yet, these clones yielded high amounts of self-replicating HCV RNAs.

Subsequent studies using replicon containing cells, found a number of adaptive mutations in the HCV NS proteins, which increased RNA replication by several orders of magnitude (28-30). It was determined that through unknown mechanisms these mutations modify the activity of the viral replicase to enhance RNA replication in Huh-7 cells. Adaptive mutations were found primarily in NS3 (31, 32), and in NS5A (33-35).

Since then, several replicons have been established for different genotype 1b isolates (36, 37), and for genotypes 1a (38) and 2a (39). Surprisingly, for reasons that are still under investigation, the genotype 2a replicon (JFH-1; derived from a Japanese patient with high levels of viremia and fulminant hepatitis) replicates approximately 20-fold more efficiently than the genotype 1-derived replicons without the need of cell culture-adaptive mutations (39, 40).

## THE HOST CELL: IMPROVING HCV REPLICATION IN HIGHLY PERMISSIVE CELL LINES

Important progress has been made in the development of cell lines permissive for HCV growth. Treatment of replicon-containing Huh-7 cells with interferon alpha (IFN-α), resulted in the clearance of replicon RNA and selection of IFN-cured cells, which were designated Huh-7.5 (41). After reintroduction of the replicon, these cells supported HCV replication to much higher levels compared to that of the naïve parental Huh-7 cells. The mechanism is unknown, but several genetic lesions have been discovered in the Huh-7.5 cells (42). In addition, the level of CD81 cell surface expression in these cells was demonstrated to be a key determinant for productive viral entry (43). HCV RNA replication has now been achieved in HeLa, HEK 293, HepG2 and mouse hepatoma cell lines (44, 45), indicating that viral replication is not restricted only to hepatocytes as it was believed for many years.

## ONE MORE STEP AHEAD: DEVELOPMENT OF FULL-LENGTH GENOMIC HCV REPLICONS

The isolation of highly permissive cell lines and the identification of adaptive mutations, constituted important tools for the establishment of efficient systems that could support autonomous replication of full-length genomic replicons. Yet, none of the RNAs generated from full-length replicons could produce infectious HCV virions (38, 39, 44). One possible explanation for this deficiency may be the interference of adaptive mutations with virus production. Mutations that promote efficient RNA replication in cell culture may be deleterious for later stages in the life cycle such as particle formation or release. This hypothesis is strongly supported by the fact that cell culture adaptive mutations present in the HCV FL-Con1 genome could block infectivity when inserted into a virus genome, preventing productive infection of chimpanzees (46). Additionally, the inability of the full-length genomic replicons to produce infectious virions could be due to the bicistronic nature of the system (Figure 3C). Possibly, the EMCV sequences, while driving up the efficiency of translation, may have a negative impact on packaging the virus.

Although the availability of the replicon system had made enormous contributions and provided extremely valuable tools to study viral replication, there are several concerns about the limitations of this system. First, the inability of the replicon to produce infectious virions; second, the requirement for adaptive mutations that are not found in naturally occurring isolates and last, the attenuated phenotype that these mutations showed in chimpanzees (46).

## A SURROGATE SYSTEM TO STUDY HCV INFECTION

Major progress has been achieved with the generation of HCV pseudo-particles (HCVpp) (47, 48), which are recombinant viral particles containing a retroviral core surrounded by an envelope, bearing native HCV glycoproteins E1 and E2. HCVpp are produced by co-transfecting cells with DNA plasmids containing a full-length HCV E1 and E2, the core proteins of either the human immunodeficiency virus (HIV) or murine leukemia virus (gag-pol genes), and a packaging-competent retroviral genome carrying a reporter gene. Transfected cells were capable of secreting approximately 10^5^ assembled pseudoparticles per ml of culture supernatant (49). Successful entry into target cells can be monitored by measuring expression of the reporter gene. Various HCVpp have been developed with envelope proteins of genotypes 1a, 1b, 2a, 3a, 4a, 5a and 6a, allowing analysis of cross- and genotype-specific neutralization (50-60). It’s been shown that HCVpp closely mimics the entry and serological properties of native and cell-culture produced HCV (49, 51), and therefore they constitute an important tool for the study of virus attachment, receptor binding and fusion processes.

## A BREAKTHROUGH IN HCV RESEARCH: PRODUCTION OF INFECTIOUS VIRAL PARTICLES

A key element towards the establishment of a robust system to study the entire HCV life cycle was the finding that transfection of a consensus JFH-1 genotype 2a full-length genome into Huh-7 cells rendered viral particles without the need of adaptive mutations (58) (Figure 3D). The secreted particles were infectious for Huh-7 cells and chimpanzees, and could be neutralized by CD81-specific antibodies and immunoglobulins from patients with chronic HCV infection. However, these particles showed a limited spread in naïve Huh-7 cells and consequently, the system rendered low viral titers. Continuous passage of JFH-1 transfected Huh-7.5.1 cells (a highly permissive Huh-7-derived cell clone, that was IFN cured from HCV replicons twice) allowed a substantial increase in the production of infectious particles, rendering 10^4^-10^5^ infectious units per ml of culture supernatant (60). Still the growth and spread of the JFH-1 virus was restricted to Huh-7-derived cell lines, since other cell lines like HepG2 and HeLa, failed to become infected.

**Figure 4:**
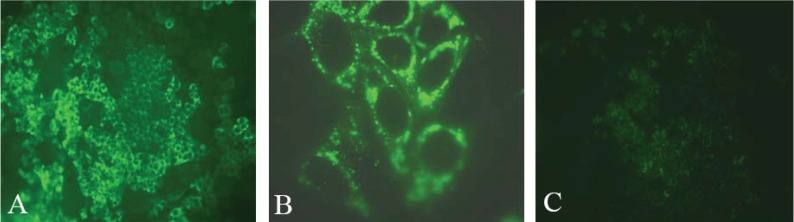
**Detection of HCV-infected cells by immunofluorescence.** Naïve Huh-7.5 cells infected with J6/JFH-1 (55) stained 3 days after infection using anti-core antibodies. (**A**) and (**B**) J6/JFH-1 Huh 7.5 infected cells; (**C**) Naïve Huh-7.5 cells. Magnification: (**A**) and (**C**): 20X; (**B**): 100 X with an oil-immersion objective.

A chimeric virus was constructed by inserting the core to NS2 region of genotype 2a HC-J6 into the JFH-1 replicon (55). Termed J6/JFH1, the virus was shown to release infectious particles from Huh-7.5 cells and produce relatively higher titers than JFH-1 (Figures 3E and 4). Subsequent studies demonstrated that chimeric J6/JFH1 viruses generated *in vitro* were infectious in chimpanzees and in a mouse model (61). Viruses recovered from these animals remained infectious in naïve Huh-7 cells. A series of chimeric genomes have since been constructed using the JFH-1 nonstructural region background, each allowing production of infectious viral particles of genotypes 1a, 1b, 2a, 3a and 4a (32, 62-64).

In addition to the JFH1-based infectious systems, several methodologies were applied to produce HCV infectious particles in cell culture. Stable human hepatoma cell lines containing a chromosomally integrated genotype 2a HCV cDNA that robustly produced HCV virions with up to 10^8^ RNA copies per ml of culture media were established (65). Furthermore, Heller *et al*. (66) constructed an infectious genotype 1b cDNA between two ribozyme structures which were designed to generate authentic HCV RNA. This system supports production of high levels of HCV virions.

Finally, a genotype 1a virus containing adaptive mutations, that was shown to infect Huh-7 cells *in vitro* (67) but to a lower infectivity than the JFH1 isolate, extends the current collection of tools available to study HCV.

## CONCLUSIONS

While all of these viruses replicate in culture, there are obvious limitations in these systems: a) they are restricted to hepatic-derived cell lines and b) only the JFH-1 isolate and JFH-1-derived viruses, robustly replicate and produce infectious virions, for reasons that are still under investigation. While JFH-1 came from a patient infected with genotype 2a virus, the reasons why this is the only strain of virus that grows well in culture remains a mystery. Still needed is a cell culture system that can utilize virus from patient serum and not from a replicon or cDNA.

Although the available systems have several restrictions and their improvement will require further investigation, it is clear that HCV can now be efficiently produced and propagated in cell culture. These cell culture systems will provide the possibility to study every step of the viral life cycle, and will contribute to the development and evaluation of new antiviral strategies to control HCV infection.

## References

[1] Hoofnagle JH (2002). Course and outcome of hepatitis C. Hepatology.

[2] Simmonds P, Bukh J, Combet C (2005). Consensus proposals for a unified system of nomenclature of hepatitis C virus genotypes. Hepatology.

[3] Chisari FV (2005). Unscrambling hepatitis C virus–host interactions. Nature.

[4] Feld JJ, Hoofnagle JH (2005). Mechanism of action of interferon and ribavirin in treatment of hepatitis C. Nature.

[5] Bartenschlager R (2002). Hepatitis C virus replicons: potential role for drug development. Nature Reviews.

[6] Reed KE, Rice CM (2000). Overview of hepatitis C virus genome structure, polyprotein processing, and protein properties. Curr. Top. Microb. Immunol.

[7] Pileri P, Uematsu Y, Campagnoli S (1998). Binding of hepatitis C virus to CD81. Science.

[8] Flint M, Loomis-Price LD, Shotton C (1999). Characterization of hepatitis C virus E2 glycoprotein interaction with a putative cellular receptor, CD81. J. Virol.

[9] Cormier EG, Tsamis F, Kajumo F (2004). CD81 is an entry coreceptor for hepatitis C virus. Proc. Natl. Acad. Sci. USA.

[10] Maillard P, Huby T, Andreo U (2006). The interaction of natural hepatitis C virus with human scavenger receptor SR-BI/Cla1 is mediated by ApoB-containing lipoproteins. FASEB J.

[11] Scarselli E, Cerino R, Roccasecca RM (2002). The human scavenger receptor class B type I is a novel candidate receptor for the hepatitis C virus. Embo. J.

[12] Evans MJ, von Hahn T, Tscherne DM (2007). Claudin-1 is a hepatitis C virus co-receptor required for a late step in entry. Nature.

[13] Agnello V, Abel G, Elfahal M (1999). Hepatitis C virus and other flaviviridae viruses enter cells via low density lipoprotein receptor. Proc. Natl. Acad. Sci. USA.

[14] Monazahian M, Bohme I, Bonk S (1999). Low density lipoprotein receptor as a candidate receptor for hepatitis C virus. J. Med. Virol.

[15] Pohlmann S, Zhang J, Baribaud F (2003). Hepatitis C virus glycoproteins interact with DC-SIGN and DC-SIGNR. J. Virol.

[16] Barth H, Ulsenheimer A, Pape GR (2005). Uptake and presentation of hepatitis C virus-like particles by human dendritic cells. Blood.

[17] van Kooyk Y, Geijtenbeek TB (2003). DC-SIGN: escape mechanism for pathogens. Nat. Rev. Immunol.

[18] Cormier EG, Durso RJ, Tsamis F (2004). L-SIGN (CD209L) and DC-SIGN (CD209) mediate transinfection of liver cells by hepatitis C virus. Proc. Natl. Acad. Sci. USA.

[19] Lozach PY, Amara A, Bartosch B (2004). C-type lectins L-SIGN and DC-SIGN capture and transmit infectious hepatitis C virus pseudotype particles. J. Biol. Chem.

[20] Tellinghuisen TL, Evans MJ, von Hahn T (2007). Studying hepatitis C virus: making the best of a bad virus. J. Virol.

[21] Pestova TV, Shatsky IN, Fletcher SP (1998). A prokaryotic-like mode of cytoplasmic eukaryotic ribosome binding to the initiation codon during internal translation initiation of hepatitis C and classical swine fever virus RNAs. Genes Dev.

[22] Lindenbach BD, Rice CM (2005). Unravelling hepatitis C virus replication from genome to function. Nature.

[23] Iacovacci S, Manzin A, Barca S (1997). Molecular characterization and dynamics of hepatitis C virus replication in human fetal hepatocytes infected *in vitro*. Hepatology.

[24] Rumin S, Berthillon P, Tanaka E (1999). Dynamic analysis of hepatitis C virus replication and quasispecies selection in long-term cultures of adult human hepatocytes infected *in vitro*. J. Gen. Virol.

[25] Nakajima N, Hijikata M, Yoshikura H (1996). Characterization of long-term cultures of hepatitis C virus. J. Virol.

[26] Deriabin PG, Viazov SO, Isaeva EI (1997). Persisitence of hepatitis C virus in newborn mice brain cell culture. Vopr. Virusol.

[27] Lohmann V, Koch J-O, Herian U (1999). Replication of subgenomic hepatitis C virus RNAs in a hepatoma cell line. Science.

[28] Blight KJ, Kolykhalov AA, Rice CM (2000). Efficient initiation of HCV RNA replication in cell culture. Science.

[29] Krieger N, Lohman V, Bartenschlager R (2001). Enhancement of hepatitis C virus RNA replication by cell culture-adaptative mutations. J. Virol.

[30] Lohmann V, Korner F, Dobierzewska A, Bartenschlager R (2001). Mutations in hepatitis C virus RNAs conferring cell culture adaptation. J. Virol.

[31] Abe K, Ikeda M, Dansako H (2007). Cell culture-adaptive NS3 mutations required for the robust replication of genome-length hepatitis C virus RNA. Virus Res.

[32] Yi M, Ma Y, Yates J, Lemon SM (2007). Compensatory mutations in E1, p7, NS2, and NS3 enhance yields of cell culture-infectious intergenotypic chimeric hepatitis C virus. J. Virol.

[33] Kohashi T, Maekawa S, Sakamoto N (2006). Site-specific mutation of the interferon sensitivity-determining region (ISDR) modulates hepatitis C virus replication. J. Viral. Hepat.

[34] Liu S, Ansari IH, Das SC, Pattnaik AK (2006). Insertion and deletion analyses identify regions of non-structural protein 5A of Hepatitis C virus that are dispensable for viral genome replication. J. Gen. Virol.

[35] Evans MJ, Rice CM, Goff SP (2004). Phosphorylation of hepatitis C virus nonstructural protein 5A modulates its protein interactions and viral RNA replication. Proc. Natl. Acad. Sci. USA.

[36] Masanori I, MinKyung Y, Kui L, Lemon SM (2002). Selectable Subgenomic and Genome-Length Dicistronic RNAs Derived from an Infectious Molecular Clone of the HCV-N Strain of Hepatitis C Virus Replicate Efficiently in Cultured Huh7 Cells. J. Virol.

[37] Zhu Q, Guo JT, Seeger C (2003). Replication of hepatitis C virus subgenomes in nonhepatic epithelial and mouse hepatoma cells. J. Virol.

[38] Blight KJ, McKeating JA, Marcotrigiano J, Rice CM (2003). Efficient replication of hepatitis C virus genotype 1a RNAs in cell culture. J. Virol.

[39] Kato T, Date T, Miyamoto M (2003). Efficient replication of the genotype 2a hepatitis C virus subgenomic replicon. Gastroenterology.

[40] Kato T, Furusaka A, Miyamoto M (2001). Sequence analysis of hepatitis C virus isolated from a fulminant hepatitis patient. J. Med. Virol.

[41] Blight K, McKeating JA, Rice CM (2002). Highly permissive cell lines for subgenomic and genomic Hepatitis C virus RNA replication. J. Virol.

[42] Sumpter R, Loo Y, Foy E (2005). Regulating intracellular antiviral defense and permissiveness to hepatitis C virus RNA replication through a cellular RNA helicase, RIG-I. J. Virol.

[43] Koutsoudakis G, Herrmann E, Kallis S (2007). The level of CD81 cell surface expression is a key determinant for productive entry of hepatitis C virus into host cells. J. Virol.

[44] Date T, Kato T, Miyamoto M (2004). Genotype 2a hepatitis C virus subgenomic replicon can replicate in HepG2 and IMY-N9 cells. J. Biol. Chem.

[45] Zhu Q, Guo JT, Seeger C (2003). Replication of hepatitis C virus subgenomes in nonhepatic epithelial and mouse hepatoma cells. J. Virol.

[46] Bukh J, Pietschmann T, Lohman V (2002). Mutations that permit efficient replication of hepatitis C virus RNA in Huh-7 cells prevent productive replication in chimpanzees. Proc. Natl. Acad. Sci. USA.

[47] Hsu M, Zhang J, Flint M (2003). Hepatitis C virus glycoproteins mediate pH-dependent cell entry of pseudotyped retroviral particles. Proc. Natl. Acad. Sci. USA.

[48] Bartosch B, Dubuisson J, Cosset FL (2003). Infectious hepatitis C virus pseudo-particles containing functional E1-E2 envelope protein complexes. J. Exp. Med.

[49] Bartosch B, Dubuisson J, Cosset FL (2003). Infectious hepatitis C virus pseudo-particles containing functional E1-E2 envelope protein complexes. J. Exp. Med.

[50] Meunier JC, Engle RE, Faulk K (2005). Evidence for cross-genotype neutralization of hepatitis C virus pseudo-particles and enhancement of infectivity by apolipoprotein C1. Proc. Natl. Acad. Sci. USA.

[51] Bartosch B, Bukh J, Meunier JC (2003). *In vitro* assay for neutralizing antibody to hepatitis C virus: evidence for broadly conserved neutralization epitopes. Proc. Natl. Acad. Sci. USA.

[52] Bartosch B, Vitelli A, Granier C (2003). Cell entry of hepatitis C virus requires a set of co-receptors that include the CD81 tetraspanin and the SR-B1 scavenger receptor. J. Biol. Chem.

[53] Lavillette D, Morice Y, Germanidis G (2005). Human serum facilitates hepatitis C virus infection, and neutralizing responses inversely correlate with viral replication kinetics at the acute phase of hepatitis C virus infection. J. Virol.

[54] Lavillette D, Tarr AW, Voisset C (2005). Characterization of host-range and cell entry properties of the major genotypes and subtypes of hepatitis C virus. Hepatology.

[55] Lindenbach BD, Evans MJ, Syder AJ (2005). Complete replication of hepatitis C virus in cell culture. Science.

[56] Logvinoff C, Major ME, Oldach D (2004). Neutralizing antibody response during acute and chronic hepatitis C virus infection. Proc. Natl. Acad. Sci. USA.

[57] McKeating JA, Zhang LQ, Logvinoff C (2004). Diverse hepatitis C virus glycoproteins mediate viral infection in a CD81-dependent manner. J. Virol.

[58] Wakita T, Pietschmann T, Kato T (2005). Production of infectious hepatitis C virus in tissue culture from a cloned viral genome. Nat. Med.

[59] Zhang J, Randall G, Higginbottom A (2004). CD81 is required for hepatitis C virus glycoprotein-mediated viral infection. J. Virol.

[60] Zhong J, Gastaminza P, Cheng G (2005). Robust hepatitis C virus infection *in vitro*. Proc. Natl. Acad. Sci. USA.

[61] Lindenbach BD, Ploss A, Vanwolleghem T (2006). Cell culture-grown hepatitis C virus is infectious *in vivo* and can be recultured *in vitro*. Proc. Natl. Acad. Sci. USA.

[62] Gottwein JM, Scheel TK, Hoegh AM (2007). Robust hepatitis C genotype 3a cell culture releasing adapted intergenotypic 3a/2a (S52/JFH1) viruses. Gastroenterology.

[63] Pietschmann T, Kaul A, Koutsoudakis G (2006). Construction and characterization of infectious intragenotypic and intergenotypic hepatitis C virus chimeras. Proc. Natl. Acad. Sci. USA.

[64] Scheel TK, Gottwein JM, Jensen TB (2008). Development of JFH1-based cell culture systems for hepatitis C virus genotype 4a and evidence for cross-genotype neutralization. Proc. Natl. Acad. Sci. USA.

[65] Cai Z, Zhang J, Chang KS (2005). Robust production of infectious hepatitis C virus (HCV) from stably HCV cDNA-transfected human hepatoma cells. J. Virol.

[66] Heller T, Saito S, Auerbach J (2005). An *in vitro* model of hepatitis C virion production. Proc. Natl. Acad. Sci. USA.

[67] Yi M, Villanueva RA, Thomas DL (2006). Production of infectious genotype 1a hepatitis C virus (Hutchinson strain) in cultured human hepatoma cells. Proc. Natl. Acad. Sci. USA.

